# Evolution of Cardiovascular Risk Factors in a Worker Cohort: A Cluster Analysis

**DOI:** 10.3390/ijerph18115610

**Published:** 2021-05-24

**Authors:** Sara Castel-Feced, Lina Maldonado, Isabel Aguilar-Palacio, Sara Malo, Belén Moreno-Franco, Eusebio Mur-Vispe, José-Tomás Alcalá-Nalvaiz, María José Rabanaque-Hernández

**Affiliations:** 1 Department of Preventive Medicine and Public Health, University of Zaragoza, 50009 Zaragoza, Spain; iaguilar@unizar.es (I.A.-P.); smalo@unizar.es (S.M.); mbmoreno@unizar.es (B.M.-F.); rabanake@unizar.es (M.J.R.-H.); 2 Fundación Instituto de Investigación Sanitaria de Aragón (IIS Aragón), 50009 Zaragoza, Spain; 3 GRISSA Research Group, 50009 Zaragoza, Spain; 4 Department of Economic Structure, Economic History and Public Economics, University of Zaragoza, 50005 Zaragoza, Spain; lmguaje@unizar.es; 5 Prevention Department, Stellantis Spain, 50639 Figueruelas, Spain; eusebio.mur@stellantis.com; 6 Department of Statistical Methods, University of Zaragoza, 50005 Zaragoza, Spain; jtalcala@unizar.es; 7 Institute of Mathematics and Applications (IUMA), 50009 Zaragoza, Spain

**Keywords:** longitudinal study, cluster analysis, real-world data

## Abstract

The identification of the cardiovascular risk factor (CVRF) profile of individual patients is key to the prevention of cardiovascular disease (CVD), and the development of personalized preventive approaches. Using data from annual medical examinations in a cohort of workers, the aim of the study was to characterize the evolution of CVRFs and the CVD risk score (SCORE) over three time points between 2009 and 2017. For descriptive analyses, mean, standard deviation, and quartile values were used for quantitative variables, and percentages for categorical ones. Cluster analysis was performed using the Kml3D package in R software. This algorithm, which creates distinct groups based on similarities in the evolution of variables of interest measured at different time points, divided the cohort into 2 clusters. Cluster 1 comprised younger workers with lower mean body mass index, waist circumference, blood glucose values, and SCORE, and higher mean HDL cholesterol values. Cluster 2 had the opposite characteristics. In conclusion, it was found that, over time, subjects in cluster 1 showed a higher improvement in CVRF control and a lower increase in their SCORE, compared with cluster 2. The identification of subjects included in these profiles could facilitate the development of better personalized medical approaches to CVD preventive measures.

## 1. Introduction

Cardiovascular diseases (CVD) are the leading cause of death worldwide [1], and result in disability and considerable economic costs on healthcare systems [2]. The global burden of disease (GBD) study in 2019 [2] showed that, during the last three decades, the prevalence of total CVD has nearly doubled, the number of CVD deaths has increased by more than 6 million and that the years of living with disability has doubled during this period. The most common CVDs are coronary heart disease and stroke [1,2]. 

Hypertension, dyslipidemia, diabetes mellitus, and smoking are well known as cardiovascular risk factors (CVRF) [3]. Obesity, another CVRF that affects children, adolescents, and adults, has doubled in prevalence over the last 3 decades [4], and numerous studies have associated abdominal obesity with insulin resistance and an increased risk of CVD [5]. 

A recent study performed in countries with different economic levels [6] has shown that approximately 70% of CVD events and deaths could be attributed to modifiable risk factors. Moreover, tobacco was the most associated with CVD among the behavioral risk factors, and hypertension among the metabolic risk factors, followed by diabetes.

CVD morbidity and mortality can be significantly reduced by preventive strategies that target both high-risk individuals and the general population [7]. Since the 1970s, a variety of population-level programs have been implemented in developed countries to promote lifestyle changes that can reduce the incidence of CVRFs [7]. A systematic review of the effectiveness of preventive programs reported different patterns of CVRF incidence, depending on the study consulted, although most of the studies included in the review indicated a positive trend [8]. Other studies of a population-level program implemented in Europe reported that after implementation of the program there was an increase in the frequency of some CVRFs, including body mass index (BMI) and glucose levels, and in physical activity, while the prevalence of hypercholesterolemia decreased [7].

Different methods are used to characterize CVD patient profiles, including so-called unsupervised learning cluster analysis, whereby individuals are grouped according to similarities in variables of interest [9]. Identification of patient profiles based on CVRFs and the risk of developing CVD can help to identify new prevention strategies, as well as identifying new lines of research. However, a study of the association between CVRFs and CVD occurrence is complicated by the fact that exposure to CVRFs might not remain constant throughout life. In this sense, the analysis of their evolution can also help in the development and improvement of prevention strategies for control of the CVRFs associated with the profiles identified. For this analysis, longitudinal data are used, where each variable is measured more than once. By clustering the trajectories of the variables into groups with similar characteristics, it is possible to convert multiple continuous variables into a single categorical variable [10], and this facilitates the classification of subjects and the subsequent analysis.

The Aragon Worker’s Health Study (AWHS) is a longitudinal cohort study designed to evaluate the evolution of CVRFs and their association with the incidence of CVD in a middle-aged population of factory workers in Spain [11]. In the present study, we hypothesized that the individuals included in the cohort will gather in different groups according to their CVRF, and that both their CVRFs and the CVD risk score will increase with time. Therefore, we sought to characterize the profiles of participants in the AWHS cohort and the evolution of CVRFs and cardiovascular risk over three time points between 2009 and 2017. 

## 2. Materials and Methods 

### 2.1. Study Design and Participants

This study was conducted within the framework of the AWHS, a prospective, longitudinal cohort study of workers at an automobile assembly plant located in Figueruelas (Zaragoza, Spain). Recruitment began in February 2009 and ended in December 2010. For the purpose of our analyses, we selected data collected at annual medical and blood tests that AWHS participants underwent at 3 different time points.

The different time points for which data were extracted are described below. Time point 1 corresponded to the first data record available for each individual after providing informed consent (i.e. data collected in 2009, 2010 or 2011, depending on when the consent form was signed and on the data availability); time point 2 corresponded to the middle of the study (2014); and time point 3 corresponded to the last data record available for each participant (it prioritized data from the year 2017, but if this was not available, data were selected from the year 2016).

**Figure 1:**
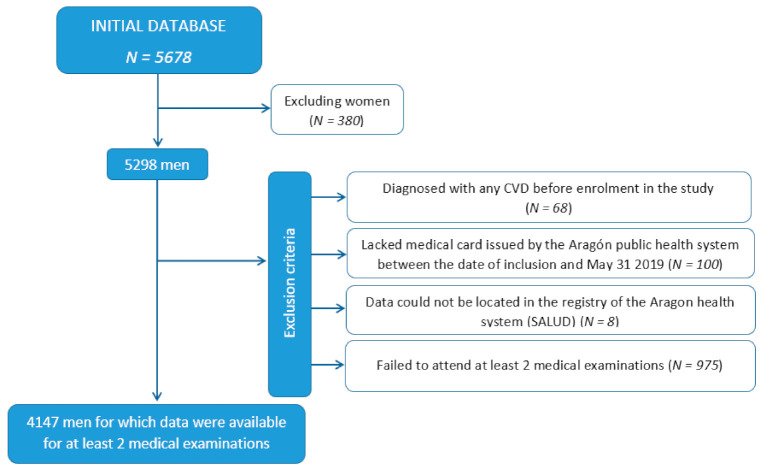
Flowchart depicting the study population.

Figure 1 depicts how the study population was selected. Our analysis was limited to men, owing to the limited number of women (*N* = 380) in the cohort. Workers diagnosed with any CVD before inclusion in the AWHS, and those who lacked a medical card issued by the Aragón public health system between the date of inclusion and 31 May 2019, were excluded from our analysis. Eight individuals for whom data could not be located in the registry of the Aragon health system (SALUD) were also excluded. 

From those selected, medical test results were not available for every year, and therefore the number of workers for which data were acquired for each time point differed. Data were available for 5122 workers for time point 1, 3891 for time point 2, and 3545 for time point 3. Finally, we excluded individuals who had not attended at least 2 of the 3 medical tests from which data were acquired for the present study (*N* = 975). The final study population consisted of 4147 individuals.

### 2.2. Variables and Data Collection

Data on BMI, waist circumference (WC), high-density lipoprotein (HDL) cholesterol, and glucose levels, collected at the annual medical examination undergone by all workers at the factory, were obtained from the AWHS database. These data were collected by the physicians and nurses of the factory’s medical services, all of whom received prior training. All study procedures were standardized. We also explored the incidence of major CVD events from the CMBD and hospital emergencies databases from the start date of the cohort study until 2017.

At each annual exam, study participants provided a clinical history and underwent a physical exam, including anthropometry (height, weight, and WC). For laboratory analyses, workers provided a sample of blood and urine after overnight (>8 hours) fasting. These samples were processed on the day of the extraction. Systolic blood pressure (SBP) and diastolic blood pressure (DBP) were recorded as quantitative variables after being measured 3 consecutive times using an automatic sphygmomanometer, with the participant sitting after a 5-minute rest. Height, weight, and WC were objectively recorded as quantitative variables. BMI was calculated from height and weight. Levels of fasting serum glucose, cholesterol, and HDL cholesterol were measured by spectrophotometry and recorded as quantitative variables in mg/dL. 

Smoking status was self-reported (current smoker, ex-smoker, or non-smoker) and was recorded as a qualitative variable. Because smoking status data were not available for time point 3, a projection procedure was applied. To this end, a transition probability matrix for smoking status was generated based on the data available for time points 1 and 2, and used to estimate the smoking status of each worker for time point 3, assuming stationarity in smoking behavior. 

Data on physical activity and alcohol intake could not be taken into account in the study, since it was neither recorded for all the subjects included in the cohort nor for all time points. Furthermore, the correlation between alcohol intake and smoking status was analyzed, and they were highly correlated.

The 10-year CVD risk score (SCORE) was estimated based on the systematic coronary risk evaluation for a European population with low cardiovascular risk [12]. To calculate this variable, smoking status was taken into account, and workers divided into 2 groups: (i) current smokers and (ii) non-smokers and ex-smokers.

Descriptive analyses were performed, taking into account quantitative variables including age, SBP, DBP, weight, WC, BMI, blood levels of HDL cholesterol, total cholesterol, and blood glucose, and SCORE, as well as some categorical variables (smoking status and group BMI).

Cluster analysis was performed, taking into account 4 CVRFs (BMI, WC, HDL cholesterol, and glucose levels), age, and the 10-year SCORE. These CVRFs were specifically selected for analysis because they were not used in the calculation of SCORE.

### 2.3. Analysis

The description of the variables was carried out using the mean and standard deviation (SD) for quantitative variables, and percentages for categorical variables. A correlation analysis of the variables included in the cluster analysis was also performed. The evolution over the study period of the quantitative variables included in the cluster analysis was analyzed using means and quartiles.

Clustering techniques are a form of unsupervised learning that gather elements in homogeneous groups based on similarities between them. Our study was a longitudinal cohort study in which each variable was measured at different time points and changed over time for each individual. The standard method of clustering variable trajectories is to cluster each variable separately. In studies involving more than one variable, cluster analysis enables analysis of the joint evolution of the variables of interest [13].

We applied the Kml3D package in R statistical software [10,14]. This k-means algorithm uses a generalized notion of distance between individual trajectories, and was used to group individuals according to the evolution of CVRF and estimated SCORE over 3 distinct time points. The Calinski–Harabasz criterion was used to determine the number of groups into which participants should be divided. Implementation of the kml3d algorithm requires that data for all variables included in the analysis be available for all participants. Because some workers did not attend all medical tests, some of these variables had to be imputed before applying the algorithm [10,15]. This was achieved using the imputation function in the R longitudinalData package [16], specifically using the “linearInterpol.bisector” command, which differentiates between monotone and intermittent missing data. In the first case, it creates the bisector of (i) the line joining the two first or the two last non-missing data (depending on whether the missing data gathers at the start or at the end of the study period) and (ii) the line joining the first and last non-missing data. In the second case, it creates a line joining the values immediately surrounding the missing value. Table 1 shows the means of the study variables calculated with both available and imputed data. They showed that the imputation worked well, since the mean variables did not change excessively. Successful imputation was confirmed by descriptive analyses and comparison of means using a Student’s t-test.

All analyses were performed using RStudio and R version 4.0.2, R Foundation for Statistical Computing, Vienna, Austria (22 June 2020). 

### 2.4. Ethical Issues

Individuals who participated in the AWHS provided prior written informed consent, and all collected data were anonymized. The present study was approved by the Clinical Research Ethics Committee of Aragon; it has not been previously conducted, and current results are not overlapped with other previously published or ongoing reports.

## 3. Results

A descriptive analysis of the cohort data for the three time points studied is shown in Table 2. 

The correlation between variables was analyzed for each time point. At time point 1, the lowest correlation indices were for WC and HDL cholesterol (−0.21) and for HDL cholesterol and BMI (−0.21). The highest correlation index (0.87) was obtained for WC and BMI. Similar results were obtained for the other 2 time points. The correlation index was significantly different from 0 for all variable pairs, except for HDL cholesterol and SCORE at time point 1, and HDL cholesterol and age at time points 2 and 3.

At the first time point analyzed, the mean weight was 81.64 kg, over half the study population was overweight, and 3 individuals included in the normal-weight group were underweight. Mean values were all within the recommended range.

Comparison of data collected at time point 2 revealed little change in mean values relative to time point 1. The greatest change observed was in the mean total cholesterol level, which was lower at time point 2 versus time point 1. The mean BMI values were practically the same at both time points. Analysis of smoking status revealed an increase in the percentage of ex-smokers and a decrease in the number of smokers and non-smokers. 

Comparison of time point 3 versus time point 2 revealed changes in the mean total cholesterol and glucose levels, both of which were decreased. 

Analysis of the overall evolution of the variables of interest over the three time points revealed a reduction in total cholesterol and glucose levels, and in smoking, and an increase in obesity.

The results of quartile analysis over the three time points for some variables are shown in Appendix A (Table A1). For time point 1, quartile analysis of BMI showed that obese workers fell in quartile (Q) 4, those with normal weight, in Q1, and those who were overweight, in Q2 and Q3. For WC and blood glucose, workers with levels above the recommended values fell in Q4, and for HDL cholesterol, those with lower than recommended levels fell in Q1.

For BMI and WC, over 80% of workers who were in Q4 at time point 1 remained in this quartile at time point 2. A similar proportion remained in Q4 between time points 2 and 3. For blood glucose levels, approximately 50% of participants who were in Q4 at time point 1 remained in this quartile at time point 2, and a similar effect was observed comparing time points 2 and 3. For HDL cholesterol levels, the percentage of workers who remained Q1 was 60% at time point 2 versus time point 1, and 80% at time point 3 versus time point 2. For SCORE, the percentage of workers who remained in the same quartile at time point 2 versus time point 1 was 70% for Q1, and 89% for Q4 (risk score, <0.63 and >2.13, respectively). For time point 3 versus time point 2, the percentage of individuals remaining in Q1 was 84%, and 88% for Q4.

**Figure 2:**
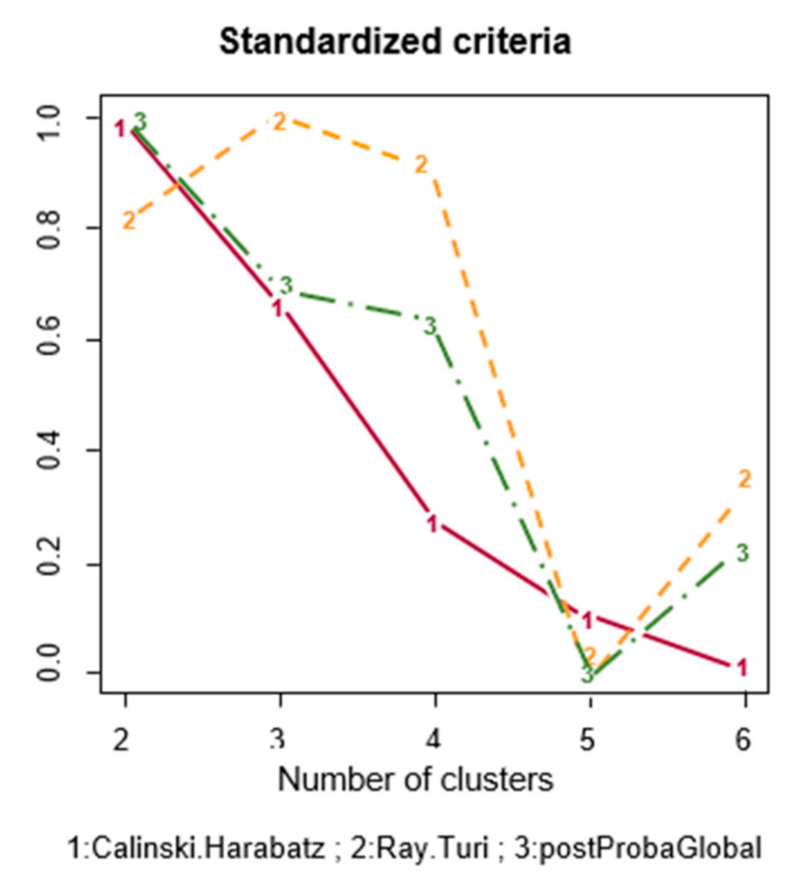
Quality index according to the number of clusters. Each line represents a different quality index, and depicts the resulting changes according to the number of clusters. Each quality index has been normalized to a value between 0 and 1.

A cluster analysis was performed to evaluate the joint evolution of the following variables: age, waist circumference, BMI, blood glucose, HDL cholesterol levels, and 10-year SCORE. Based on the quality index to determine the number of clusters (Figure 2), we divided the cohort into 2 and 3 clusters. When analyses were performed for both scenarios, results were better justified when the cohort was divided into 2 rather than 3 groups. Moreover, the Calinski–Harabasz score was 1197 when the cohort was divided into 2 clusters, and 1067 when divided into 3. Therefore, we focused on the results obtained using 2 clusters.

**Figure 3:**
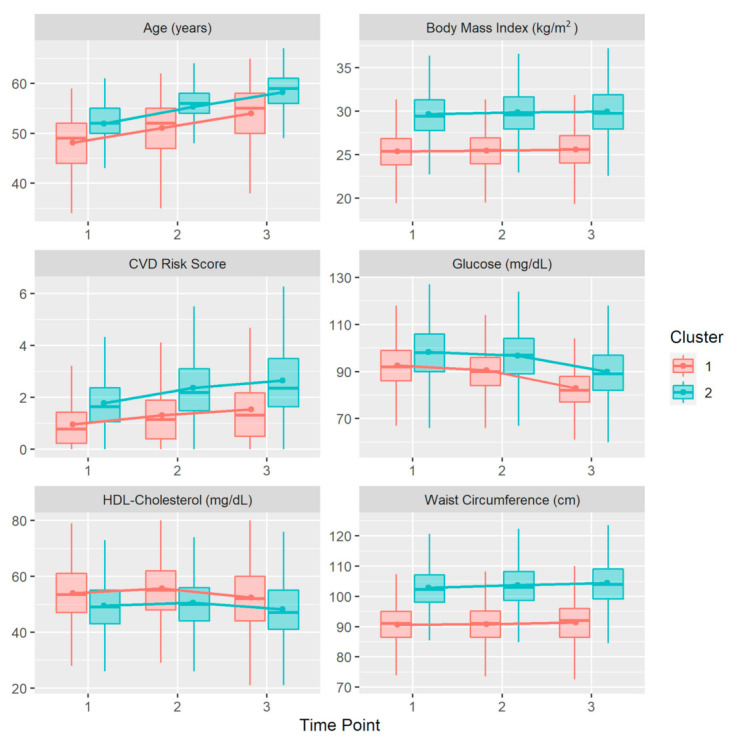
Box plots depicting mean values (dots) for each variable per cluster. Lines represent the evolution of the mean for each cluster over the three time points analyzed. Abbreviation: CVD, cardiovascular disease.

Figure 3 shows the mean values obtained for each variable, stratified by clusters. Table 3 depicts the descriptive analysis of the cohort, stratified by cluster and time point. 

Cluster 1 consisted of younger workers with a lower mean BMI, WC, blood glucose values, and SCORE, and higher mean HDL cholesterol values. Analysis of the evolution of each variable over the three time points revealed similar patterns in both clusters. The mean WC and BMI values increase slightly and progressively over time, although this increase was slightly greater in cluster 2. Blood glucose levels decreased slightly and progressively over time in both clusters. In both clusters, HDL cholesterol levels increased between time points 1 and 2 and decreased between time points 2 and 3. Finally, in both clusters, SCORE increased over the study period, although this effect was greater in cluster 2.

The evolution per cluster of each quartile over the three time points is shown in Appendix A (Table A2). For BMI, in both clusters 1 and 2, more than half of the individuals in a given BMI quartile at time point 1 remained in the same quartile at time point 2, with the exception of Q4. The percentage of individuals who remained in Q4 at time point 2 was twice as high in cluster 2 versus cluster 1. Moreover, in cluster 1, the percentage of individuals in Q4 who remained in this quartile at time point 2 was similar to the percentage that switched to Q3.

Comparison of BMI values at time points 2 and 3 revealed similar findings to the comparison of time points 1 and 2 in both clusters. However, in cluster 1, the percentage of subjects who remained in Q4 between time points 2 and 3 was greater than the percentage of subjects who remained in Q4 between time points 1 and 2. The evolution of WC was very similar to that of BMI.

For HDL cholesterol, in cluster 1, 54% of workers who were in Q1 at time point 1 remained in this quartile at time point 2. The percentage of workers in Q1 at both time points 2 and 3 was 78%. In cluster 2, 65% of workers in Q1 at time point 1 remained in this quartile at time point 2. In this same cluster, the percentage of workers in Q1 at both time points 2 and 3 was 82%. 

Analysis of blood glucose values showed that in cluster 2, the proportion of workers who were at Q4 at time point 1 and remained in this quartile at time point 2 was double that observed for cluster 1. For both clusters, between time points 2 and 3, we observed a decrease in the proportion of workers that did not switch quartiles, except for Q1, for which a significant increase was observed.

Finally, for SCORE, in cluster 1 the percentage of workers who were in Q1 and Q4 at both time points 1 and 2 (74% and 82%, respectively) was higher than that observed for Q2 and Q3 (38% and 48%, respectively). Comparison of time points 2 and 3 showed that for all quartiles, the percentage of workers who did not switch quartile was higher than that observed between time points 1 and 2. In cluster 2, the results obtained were similar to those observed for cluster 1, although among workers in Q1 at time point 1, only 41% remained in this quartile at time point 2, whereas 46% moved to Q2. 

In an initial study about CVD events, it was found that of the 45 individuals who suffered a major CVD event, 13 belonged to cluster 1, and 32 to cluster 2.

## 4. Discussion

In this study, we analyzed the different trajectories of CVRFs and SCORE in a cohort of factory workers across three time points. The quantitative variables that underwent the greatest changes over the entire study period were total cholesterol, glucose, and SCORE. The mean SCORE increased, while the mean total cholesterol and glucose values decreased. These results are in line with those of our quartile analysis. It should be borne in mind, first, that the observed increase in SCORE could be mainly due to the increasing age. Second, the assessments of the individuals included in the cohort are periodically followed up intensively by the factory medical services. Thus, the decrease in the total cholesterol and glucose levels, and the stabilization of blood pressure could be due to the close control of these patients, as these CVRFs are managed through pharmacological treatment. The BMI did not show significant changes, although more than half of all participants were overweight for the duration of the study. Another noteworthy finding was the decrease over time in the percentage of smokers. 

Different authors [12,17] have reported the influence of age on calculating SCORE. Conroy et al. [12] found that SCORE was very low in people aged 30, and that it increased most rapidly between 50 and 65 years. In the present study, SCORE increased over time from 1.56 to 2.09. This increase was probably lower than expected, which may be due to the effect of the stabilization and improvement of some CVRFs. Thus, glucose and total cholesterol mean levels decreased from 97.7 mg/dl to 88.06 mg/dl, and from 212.18 mg/dl to 187.96 mg/dl, respectively, between the first and last time points, and the BMI mean remained stable over the three time points (27.61 at the first, versus 27.84 at the third).

To create clusters, we used an algorithm that divided the study cohort into groups based on the trajectories of different variables over time. This algorithm was applied to the mean CVRF values and SCORE over the three time points analyzed. The results showed that study participants could be divided into 2 clusters, based on the evolution of CVRF values and SCORE. 

The first cluster consisted of younger individuals with lower mean blood glucose, BMI, and WC values, higher SCORE values, and higher mean HDL cholesterol values. The second cluster consisted of older individuals with higher mean blood glucose, BMI, and WC values, higher SCORE, and lower mean HDL cholesterol values.

The analysis of changes in quartiles measured by clusters revealed that the percentage of individuals who remained in quartiles with higher than recommended BMI, WC, and glucose values, and lower than recommended HDL cholesterol values, was higher in cluster 2 than in cluster 1. Furthermore, in both clusters this percentage increased over time for all variables except glucose, for which decreases over the three time points were observed. For SCORE, a similar evolution was observed in both clusters for individuals who began the study in Q2, Q3 or Q4. The greatest difference between clusters was observed for workers with a SCORE in Q1: the proportion of workers who remained in this quartile over the three time points was greater in cluster 1 than in cluster 2.

Few published studies have used clustering methodology similar to ours to analyze the evolution of CVRFs. Several studies have analyzed the trajectories of one [18,19,20,21] or several [22,23] CVRFs using a variety of different methods, and have attempted to identify correlations between their findings and other factors or diseases. One such study [19] analyzed the evolution of SBP, for which 4 distinct trajectories were identified over time. The authors found that SBP trajectories predicted CVD and all-cause mortality no better than did mean SBP values. Another study [21] of SBP and DBP in an elderly population identified 3 blood pressure (BP) trajectories. BP trajectories were also analyzed by Allen et al. [20], who identified 5 BP trajectories in a middle-aged population. Rospleszcz et al. [24] analyzed the association between CVRF trajectories and adipose tissue deposits using a methodology similar to ours and identified 3 distinct clusters. The first cluster grouped individuals with the youngest mean age and lowest mean CVRF values, and the third cluster grouped those with the highest mean age and highest mean CVRF values. Mean age and mean CVRF values in cluster 2 fell between those of clusters 1 and 3, except for total and HDL cholesterol, for which values were higher than the other 2 clusters. Finally, Norby et al. [22] used a mixture model to separately identify the trajectories of different CVRFs. Five distinct trajectories were identified for BMI, obesity, and SBP, and 4 for hypertension and diabetes. 

Although the analysis of the incidence of CVD events was preliminary, more events were detected in cluster 2 than in cluster 1.

Our study has some limitations. First, the study population was exclusively male, owing to the low number of women in the AWHS cohort. Although it is not representative of the general population, it represents workers well in this type of factory and in these age ranges, which represents an important part of the population. Second, despite the large study population, not all participants attended each of the 3 medical tests for which the study data were extracted. Moreover, the algorithm used does not tolerate missing data, and therefore we were obliged to impute data in some cases and eliminate data in cases in which imputation was not possible. Third, the selected study period was relatively short for a trajectory study. Fourth, due to the young age of the workers, the number of events detected in the preliminary study of the incidence of CVD events was low. Several strengths of our study should also be noted. The methodology used is based on a k-means clustering algorithm that can simultaneously consider different variables and time points (i.e. trajectories of multiple variables). This study presents clusters according to the evolution of different CVRFs at the same time, in contrast to previous studies aimed at creating clusters according to each CVRF separately. Furthermore, the data analyzed were extracted from multiple sources, and included multiple well-refined variables. Nevertheless, further studies conducted over longer time periods will be required to evaluate differences between clusters in the incidence of CVD events. 

Finally, regarding the worker cohort, these results could help the development of personalized medicine by the factory medical services, to improve the cardiovascular health of the workers and to implement preventive measures. 

## 5. Conclusions

Using clustering analysis, we found that our cohort could be divided into 2 groups. The profile of cluster 1 was a lower age, BMI, WC, blood glucose level, and SCORE, and higher HDL cholesterol levels. Cluster 2 consisted of individuals with higher BMI, WC, blood glucose levels, and SCORE, and lower HDL cholesterol levels. Finally, although no significant changes in CVRFs were detected during the study period, the worsening of CVRFs was greater in cluster 2 than in cluster 1. Individuals in both groups increased their SCORE, but this increase was greater in the ones in cluster 2, as their worsening of CVRFs was higher. Thus, the identification of subjects included in these profiles could facilitate the development of better, personalized medicine approaches to CVD treatment and preventive measures, especially in those profiles showing the worst CVRF control.

## Figures and Tables

**Table 1:** Comparison between real and imputed data.

Variables	Time Point 1		Time Point 2		Time Point 3	
Data	Imputed	Real	Imputed	Real	Imputed	Real
BMI (kg/m**^2^**)	27.6 (3.5)	27.6 (3.5)	27.8 (3.7)	27.8 (3.7)	27.9 (3.8)	27.8 (3.8)
Wc- Cholesterol (cm)	96.8 (9.7)	96.8 (9.6)	97.4 (10.0)	97.3 (10.0)	98.1 (10.8)	97.7 (10.5)
HDL (mg/dL)	52.4 (10.9)	52.4 (11.0)	53.8 (11.3)	54.1 (11.3)	51.5 (12.9)	51.0 (12.4)
Glucose (mg/dL)	97.7 (18.7)	97.7 (18.7)	98.4 (19.5)	96.5 (19.5)	89.9 (21.5)	88.1 (18.6)
SCORE	1.6 (1.4)	1.6 (1.4)	2.1 (1.7)	2.1 (1.7)	2.4 (2.2)	2.1 (1.7)

Abbreviations: WC, waist circumference; BMI, body mass index; SCORE, cardiovascular disease risk score.

**Table 2:** Descriptive analysis of the study variables.

Quantitative Variables		Time Point 1 Mean (SD)	Time Point 2 Mean (SD)	Time Point 3 Mean (SD)
Age (years)		48.00 (8.42)	51.49 (8.27)	53.00 (8.25)
Systolic blood pressure (mmHg)		126.00 (14.14)	124.00 (14.25)	128.89 (15.00)
Diastolic blood pressure (mmHg)		83.44 (9.82)	79.80 (9.39)	81.36 (9.68)
Weight (kg)		81.64 (11.47)	82.10 (11.92)	82.66 (12.38)
Waist circumference (cm)		96.81 (9.61)	97.30 (10.00)	97.73 (10.53)
Body mass index (kg/m^2^)		27.61 (3.54)	27.77 (3.67)	27.84 (3.80)
HDL cholesterol (mg/dL)		52.45 (11.00)	54.07 (11.30)	51.00 (12.40)
Total cholesterol (mg/dL)		212.18 (37.62)	205.93 (34.75)	187.96 (32.85)
Glucose (mg/dL)		97.70 (18.75)	96.51 (19.46)	88.06 (18.60)
SCORE		1.56 (1.40)	2.05 (1.73)	2.09 (1.74)
**Categorical Variables**		**Time Point 1** ***N* (%)**	**Time Point 2** ***N* (%)**	**Time Point 3** ***N* (%)**
**Smoking status**	Smoker	1488 (36.82)	1235 (32.19)	1156 (32.65)
	Non-smoker	1087 (26.90)	925 (24.11)	853 (24.09)
	Ex-smoker	1466 (36.28)	1677 (43.71)	1532 (43.26)
**Body mass index groups**	Normal weight	938 (23.05)	846 (22.01)	762 (22.38)
	Overweight	2223 (54.63)	2088 (54.33)	1813 (54.25)
	Obesity	908 (22.32)	909 (23.65)	830 (24.38)

Abbreviations: SD, standard deviation; SCORE, cardiovascular disease risk score; *N*, number. Smoking status data for the time point 3 were estimated by imputation.

**Table 3:** Descriptive analysis of the variables included in the cluster analysis, stratified by cluster and time point.

Variables	Time Point	Cluster 1 N = 2099	Cluster 2 N = 2048	*p*
		Mean (SD)	Mean (SD)	
**Age**	Time point 1	44.2 (9.58)	51.7 (4.59)	<0.001
	Time point 2	47.6 (9.58)	55.2 (4.63)	<0.001
	Time point 3	50.6 (9.55)	58.1 (4.58)	<0.001
**WC**	Time point 1	90.5 (6.80)	103 (7.72)	<0.001
	Time point 2	90.6 (6.61)	104 (8.06)	<0.001
	Time point 3	91.2 (7.31)	105 (8.97)	<0.001
**BMI**	Time point 1	25.3 (2.30)	30.0 (3.04)	<0.001
	Time point 2	25.4 (2.25)	30.2 (3.22)	<0.001
	Time point 3	25.6 (2.41)	30.4 (3.49)	<0.001
**Glucose**	Time point 1	92.9 (11.7)	103.0 (22.9)	<0.001
	Time point 2	91.0 (11.5)	102.0 (24.1)	<0.001
	Time point 3	83.4 (11.7)	96.5 (26.7)	<0.001
**HDL**	Time point 1	55.0 (11.3)	49.9 (9.94)	<0.001
	Time point 2	56.8 (11.8)	50.9 (9.96)	<0.001
	Time point 3	54.1 (13.4)	48.9 (11.8)	<0.001
**SCORE**	Time point 1	1.02 (1.03)	2.12 (1.55)	<0.001
	Time point 2	1.38 (1.27)	2.74 (1.85)	<0.001
	Time point 3	1.62 (1.49)	3.27 (2.49)	<0.001

Abbreviations: WC, waist circumference; BMI, body mass index; SCORE, cardiovascular disease risk score; *p*, *p*-value (unpaired 2-sample Student’s t-test).

## Data Availability

The data presented in this study are available on request from the corresponding author. The data are not public due to ethical reasons.

## Appendix A

**Table A1:** Results of the quartile analysis. Values represent the percentage of individuals that moved from one quartile to another between time points 1 and 2 and time points 2 and 3 for the following variables: body mass index, blood glucose, and cardiovascular disease risk score.

BODY MASS INDEX													
		**Time Point 1**							**Time Point 2**				
		Q. 1	Q. 2	Q. 3	Q. 4	*p*			Q. 1	Q. 2	Q. 3	Q. 4	*p*
		N (%)	N (%)	N (%)	N (%)				N (%)	N (%)	N (%)	N (%)	
**Time point 2**	Q. 1	814 (79.0%)	143 (13.8%)	15 (1.4%)	2 (0.2%)	<0.001	**Time point 3**	Q. 1	819 (84.1%)	156 (14.8%)	11 (1.07%)	1 (0.1%)	<0.001
	Q. 2	203 (19.7%)	660 (63.9%)	182 (17.3%)	10 (1.0%)			Q. 2	140 (14.4%)	679 (64.4%)	155 (15.1%)	9 (0.8%)	
	Q. 3	14 (1.4%)	221 (21.4%)	658 (62.7%)	134 (13.0%)			Q. 3	13 (1.3%)	214 (20.3%)	678 (66.0%)	109 (10.0%)	
	Q. 4	0 (0.0%)	9 (0.9%)	194 (18.5%)	888 (85.9%)			Q. 4	2 (0.2%)	6 (0.6%)	183 (17.8%)	972 (89.1%)	
**BLOOD GLUCOSE**													
		**Time point 1**							**Time point 2**				
		Q. 1	Q. 2	Q. 3	Q. 4	*p*			Q. 1	Q. 2	Q. 3	Q. 4	*p*
		N (%)	N (%)	N (%)	N (%)				N (%)	N (%)	N (%)	N (%)	
**Time point 2**	Q. 1	713 (64.0%)	356 (35.2%)	180 (16.8%)	49 (5.2%)	<0.001	**Time point 3**	Q. 1	1103 (85.0%)	790 (74.0%)	458 (45.8%)	107 (13.7%)	<0.001
	Q. 2	260 (23.3%)	351 (34.8%)	359 (33.5%)	98 (10.3%)			Q. 2	140 (10.8%)	181 (16.9%)	279 (27.9%)	113 (14.5%)	
	Q. 3	106 (9.5%)	239 (23.7%)	372 (34.7%)	282 (29.7%)			Q. 3	40 (3.1%)	72 (6.7%)	169 (16.9%)	158 (20.2%)	
	Q. 4	35 (3.1%)	64 (6.3%)	161 (15.0%)	522 (54.9%)			Q. 4	15 (1.2%)	25 (2.3%)	93 (9.3%)	404 (51.7%)	
**CARDIOVASCULAR DISEASE RISK SCORE**													
		**Time point 1**							**Time point 2 **				
		Q. 1	Q. 2	Q. 3	Q. 4	*p*			Q. 1	Q. 2	Q. 3	Q. 4	*p*
		N(%)	N(%)	N(%)	N(%)				N(%)	N(%)	N(%)	N(%)	
**Time point 2**	Q. 1	722 (69.6%)	15 (1.5%)	0 (0.0%)	0 (0.0%)	<0.001	**Time point 3**	Q. 1	618 (83.9%)	28 (4.0%)	6 (0.5%)	10 (0.6%)	<0.001
	Q. 2	272 (26.2%)	360 (34.8%)	65 (6.3%)	9 (0.9%)			Q. 2	115 (15.6%)	353 (50.0%)	85 (7.5%)	18 (1.2%)	
	Q. 3	38 (3.7%)	544 (52.6%)	453 (43.8%)	108 (10.4%)			Q. 3	3 (0.4%)	296 (41.9%)	636 (55.6%)	154 (9.9%)	
	Q. 4	6 (0.6%)	116 (11.2%)	517 (50.0%)	922 (88.7%)			Q. 4	1 (0.1%)	29 (4.1%)	416 (36.4%)	1379 (88.3%)	

Abbreviations: Q, quartile; N, number; *p*, *p*-value (Chi-squared and Mann-Whitney U-test).

**Table A2:** Results of the quartile analysis by clusters. Values represent the percentage of individuals that moved from one quartile to another between time points 1 and 2 and time points 2 and 3 for the following variables: body mass index, blood glucose, and cardiovascular disease risk score.

BODY MASS INDEX													
		**Time Point 1**							**Time Point 2**				
		Q. 1 N (%)	Q. 2 N (%)	Q. 3 N (%)	Q. 4 N (%)	*p*			Q. 1 N (%)	Q. 2 N (%)	Q. 3 N (%)	Q. 4 N (%)	*p*
	**Cluster 1**					<0.001	**Time point 3**	**Cluster 1**					<0.001
**Time point 2**	Q. 1	789 (80.4%)	125 (17.2%)	10 (3.0%)	1 (1.7%)			Q. 1	786 (85.0%)	126 (16.3%)	3 (0.8%)	0 (0.0%)	
	Q. 2	179 (18.2%)	481 (66.1%)	106 (31.9%)	5 (8.6%)			Q. 2	127 (13.7%)	515 (66.8%)	76 (22.1%)	4 (6.8%)	
	Q. 3	13 (1.3%)	119 (16.3%)	187 (56.3%)	25 (43.1%)			Q. 3	11 (1.2%)	126 (16.3%)	220 (64.0%)	18 (30.5%)	
	Q. 4	0 (0.0%)	3 (0.4%)	29 (8.7%)	27 (46.6%)			Q. 4	1 (0.1%)	4 (0.5%)	45 (13.1%)	37 (62.7%)	
**Time point 2**	**Cluster 2**					<0.001	**Time point 3**	**Cluster 2**					<0.001
	Q. 1	25 (50.0%)	18 (5.9%)	5 (0.7%)	1 (0.1%)			Q. 1	33 (67.3%)	30 (10.6%)	8 (1.2%)	1 (0.1%)	
	Q. 2	24 (48.0%)	179 (58.7%)	76 (10.6%)	5 (0.5%)			Q. 2	13 (26.5%)	164 (57.7%)	79 (11.6%)	5 (0.5%)	
	Q. 3	1 (2.0%)	102 (33.4%)	471 (65.7%)	109 (11.2%)			Q. 3	2 (4.1%)	88 (31.0%)	458 (67.1%)	91 (8.8%)	
	Q. 4	0 (0.0%)	6 (2.0%)	165 (23.0%)	861 (88.2%)			Q. 4	1 (2.0%)	2 (0.7%)	138 (20.2%)	935 (90.6%)	
**BLOOD GLUCOSE**													
		**Time point 1**							**Time point 2**				
		Q. 1 N (%)	Q. 2 N (%)	Q. 3 N (%)	Q. 4 N (%)	*p*			Q. 1 N (%)	Q. 2 N (%)	Q. 3 N (%)	Q. 4 N (%)	*p*
**Time point 2**	**Cluster 1**					<0.001	**Time point 3**	**Cluster 1**					<0.001
	Q. 1	487 (67.5%)	249 (39.6%)	96 (19.5%)	27 (10.5%)			Q. 1	765 (89.1%)	520 (81.4%)	257 (58.4%)	40 (24.8%)	
	Q. 2	172 (23.9%)	234 (37.2%)	193 (39.2%)	40 (15.6%)			Q. 2	67 (7.8%)	92 (14.4%)	108 (24.5%)	37 (23.0%)	
	Q. 3	52 (7.2%)	133 (21.1%)	162 (32.9%)	93 (36.2%)			Q. 3	19 (2.2%)	23 (3.6%)	56 (12.7%)	35 (21.7%)	
	Q. 4	10 (1.4%)	13 (2.1%)	41 (8.3%)	97 (37.7%)			Q. 4	8 (0.9%)	4 (0.6%)	19 (4.3%)	49 (30.4%)	
													
**Time point 2**	**Cluster 2**					<0.001	**Time point 3**	**Cluster 2**					<0.001
	Q. 1	226 (57.5%)	107 (28.1%)	84 (14.5%)	22 (3.2%)			Q. 1	338 (77.0%)	270 (62.9%)	201 (36.0%)	67 (10.8%)	
	Q. 2	88 (22.4%)	117 (30.7%)	166 (28.6%)	58 (8.4%)			Q. 2	73 (16.6%)	89 (20.7%)	171 (30.6%)	76 (12.2%)	
	Q. 3	54 (13.7%)	106 (27.8%)	210 (36.2%)	189 (27.2%)			Q. 3	21 (4.8%)	49 (11.4%)	113 (20.2%)	123 (19.8%)	
	Q. 4	25 (6.4%)	51 (13.4%)	120 (20.7%)	425 (61.2%)			Q. 4	7 (1.6%)	21 (4.9%)	74 (13.2%)	355 (57.2%)	
**CARDIOVASCULAR DISEASE RISK SCORE**													
		**Time point 1**							**Time point 2**				
		Q. 1 N (%)	Q. 2 N (%)	Q. 3 N (%)	Q. 4 N (%)	*p*			Q. 1 N (%)	Q. 2 N (%)	Q. 3 N (%)	Q. 4 N (%)	*p*
**Time point 2**	**Cluster 1**					<0.001	**Time point 3**	**Cluster 1**					<0.001
	Q. 1	657 (74.7%)	10 (1.7%)	0 (0.0%)	0 (0.0%)			Q. 1	578 (86.7%)	19 (4.2%)	1 (0.2%)	2 (0.5%)	
	Q. 2	200 (22.7%)	215 (38.0%)	31 (7.8%)	4 (1.6%)			Q. 2	86 (12.9%)	241 (53.6%)	52 (9.4%)	3 (0.7%)	
	Q. 3	23 (2.6%)	298 (52.7%)	192 (48.2%)	40 (15.7%)			Q. 3	2 (0.3%)	177 (39.3%)	322 (58.2%)	54 (12.6%)	
	Q. 4	0 (0.0%)	43 (7.6%)	175 (44.0%)	211 (82.7%)			Q. 4	1 (0.2%)	13 (2.9%)	178 (32.2%)	370 (86.2%)	
**Time point 2**	**Cluster 2**					<0.001	**Time point 3**	**Cluster 2**					<0.001
	Q. 1	65 (41.1%)	5 (1.1%)	0 (0.0%)	0 (0.0%)			Q. 1	40 (57.1%)	9 (3.5%)	5 (0.9%)	8 (0.7%)	
	Q. 2	72 (45.6%)	145 (30.9%)	34 (5.3%)	5 (0.6%)			Q. 2	29 (41.4%)	112 (43.8%)	33 (5.6%)	15 (1.3%)	
	Q. 3	15 (9.5%)	246 (52.5%)	261 (41.0%)	68 (8.7%)			Q. 3	1 (1.4%)	119 (46.5%)	314 (53.2%)	100 (8.8%)	
	Q. 4	6 (3.8%)	73 (15.6%)	342 (53.7%)	711 (90.7%)			Q. 4	0 (0.0%)	16 (6.3%)	238 (40.3%)	1009 (89.1%)	

Abbreviations: Q, quartile; N, number; *p*, *p*-value (Chi-squared and Mann-Whitney U-test).
